# Generic semi-automated radiofluorination strategy for single domain antibodies: [^18^F]FB-labelled single domain antibodies for PET imaging of fibroblast activation protein-α or folate receptor-α overexpression in cancer

**DOI:** 10.1186/s41181-024-00286-8

**Published:** 2024-07-24

**Authors:** Herlinde Dierick, Laurent Navarro, Hannelore Ceuppens, Thomas Ertveldt, Ana Rita Pombo Antunes, Marleen Keyaerts, Nick Devoogdt, Karine Breckpot, Matthias D’Huyvetter, Tony Lahoutte, Vicky Caveliers, Jessica Bridoux

**Affiliations:** 1 Molecular Imaging and Therapy Research Group (MITH), Vrije Universiteit Brussel (VUB), Laarbeeklaan 103. Building K., 1090 Brussels, Belgium; 2 Nuclear Medicine Department, Vrije Universiteit Brussel (VUB), Universitair Ziekenhuis Brussel (UZ Brussel), Laarbeeklaan 101, 1090 Brussels, Belgium; 3 Precirix NV, Burgemeester Etienne Demunterlaan 3, 1090 Brussels, Belgium; 4 Laboratory for Molecular and Cellular Therapy (LCMT), Department of Biomedical Sciences, Translational Oncology Research Center, Vrije Universiteit Brussel (VUB), Laarbeeklaan 103. Building E, 1090 Brussels, Belgium

**Keywords:** Fluorine-18, Single domain antibodies, Automation, Biomolecules

## Abstract

**Background:**

Radiofluorination of single domain antibodies (sdAbs) via *N*-succinimidyl-4-[^18^F]fluorobenzoate ([^18^F]SFB) has shown to be a promising strategy in the development of sdAb-based PET tracers. While automation of the prosthetic group (PG) [^18^F]SFB production, has been successfully reported, no practical method for large scale sdAb labelling has been reported. Therefore, we optimized and automated the PG production, enabling a subsequently efficient manual conjugation reaction to an anti-fibroblast activation protein (FAP)-α sdAb (4AH29) and an anti-folate receptor (FR)-α sdAb (2BD42). Both the alpha isoform of FAP and the FR are established tumour markers. FAP-α is known to be overexpressed mainly by cancer-associated fibroblasts in breast, ovarian, and other cancers, while its expression in normal tissues is low or undetectable. FR-α has an elevated expression in epithelial cancers, such as ovarian, brain and lung cancers. Non-invasive imaging techniques, such as PET-imaging, using tracers targeting specific tumour markers can provide molecular information over both the tumour and its environment, which aides in the diagnosis, therapy selection and assessment of the cancer treatment.

**Results:**

[^18^F]SFB was synthesized using a fully automated three-step, one-pot reaction. The total procedure time was 54 min and results in [^18^F]SFB with a RCP > 90% and a RCY d.c. of 44 ± 4% (n = 13). The manual conjugation reaction after purification produced [^18^F]FB-sdAbs with a RCP > 95%, an end of synthesis activity > 600 MBq and an apparent molar activity > 10 GBq/µmol. Overall RCY d.c., corrected to the trapping of [^18^F]F^−^ on the QMA, were 9% (n = 1) and 5 ± 2% (n = 3) for [^18^F]FB-2BD42 and [^18^F]FB-4AH29, respectively.

**Conclusion:**

[^18^F]SFB synthesis was successfully automated and upscaled on a Trasis AllInOne module. The anti-hFAP-α and anti-hFR-α sdAbs were radiofluorinated, yielding similar RCYs d.c. and RCPs, showing the potential of this method as a generic radiofluorination strategy for sdAbs. The radiofluorinated sdAbs showed a favourable biodistribution pattern and are attractive for further characterization as new PET tracers for FAP-α and FR-α imaging.

**Supplementary Information:**

The online version contains supplementary material available at 10.1186/s41181-024-00286-8.

## Background

Both the alpha isoforms of the Folate Receptor (FR) and Fibroblast Activation Protein (FAP) are established tumour markers. FR-α has an elevated expression in epithelial cancers, such as ovarian, cervical, and head and neck cancer (Sega and Low 2008). At the same time, this isoform has a minimal physiological role in healthy tissue (except during embryogenesis), making it an interesting anticancer target. FR-α also shows a high affinity for both physiological and non-physiological substrates, which further cements its relevance for diagnostic and theranostic purposes (Scaranti et al. 2020; Boss and Ametamey 2020).

Only one FR-α targeting therapy, Mirvetuximab, Soravtansine (Moore et al. 2023), has been approved for use in patients (Harada et al. 2024). Other promising agents, such as Farletuzumab (Herzog et al. 2023) and Vintafolide (2024), failed to meet their primary endpoints. A positron emission tomography (PET) tracer that specifically targets FR-α has the potential to be a companion diagnostic for FR-α targeting therapies and can help in patient stratification (Harada et al. 2024; Guzik et al. 2021) The last decades, a large number of folate tracers, however not specifically targeting FR-α, labelled with fluorine-18 (^18^F) have been developed. To our knowledge, only two have made it to clinical trials, namely [^18^F]-AzaFol (Gnesin et al. 2020) and [^18^F]fluoro-PEG-folate (Verweij et al. 2020).

FAP is known to be overexpressed on cancer-associated fibroblasts within the tumour microenvironment of breast, colorectal, ovarian, and other cancers, while its expression is low or undetectable normal tissues (Fitzgerald and Weiner 2020). Due to this attractive expression pattern, anti-FAP radiopharmaceuticals have been a hot topic for diagnostic and therapeutic applications. Several FAP targeting small molecule compounds, for example OncoFAP (Backhaus et al. 2022), FAPI-04 (Wang et al. 2021a, 2021b), FAPI-46 (Meyer et al. 2020), FAPI-74 (Giesel et al. 2021) and PNT6555 (Poplawski et al. 2023) and peptide-based radiopharmaceuticals, such as FAP-2286 (Zboralski et al. 2022), have been developed in recent years and are currently being tested in the clinic (Zboralski et al. 2022; Millul et al. 2021; Zhao et al. 2022; Toyohara et al. 2022).

Different targeting moieties have been used to develop PET tracers for established tumour markers. Immune-derived vectors such as monoclonal antibodies (mAbs), minibodies, single-domain antibodies (sdAbs), allow to combine their highly specific targeting with the sensitivity and resolution of PET (Wei et al. 2020). SdAbs have gained quite some interest as targeting molecules for PET imaging. Their key characteristics, such as their small size (around 15 kDa), high affinity, high specificity, low off-target accumulation, high (thermo)stability and solubility (Pauw et al. 2023) allowed them to be successfully translated to the clinic as diagnostic (Keyaerts et al. 2016; Gondry et al. 2024) and therapeutic (D’Huyvetter et al. 2021) radiopharmaceuticals. Compared to mAb-based diagnostics, their most notable advantages that their short biological half-life and fast tumour penetration allow for their labelling with short-lived radionuclides such as gallium-68 (^68^Ga) and ^18^F (Pauw et al. 2023).

From a diagnostic standpoint, ^18^F is an ideal radionuclide for PET imaging with its high positron (β^+^) yield of 97%, relatively low energy (max 0.634 MeV) of the emitted β^+^ and thus short trajectory (mean positron range in soft tissue: 0.27 mm) resulting in high-resolution images. Its half-life of 109.8 min is long enough to allow shipment of the radiopharmaceutical to other centres but still short enough to avoid unnecessary extended irradiation of the patients. The ease of producing large amounts with a cyclotron cements its place as the favourite radionuclide in PET imaging (Wei et al. 2020; Cleeren et al. 2018). The direct ^18^F-labelling of sdAbs and other biomolecules is prevented by the harsh reaction conditions, elevated temperatures, organic solvents, and high pH needed for radiofluorination. The development of prosthetic groups (PG) like *N*-succinimidyl 4-[^18^F]Fluorobenzoate ([^18^F]SFB), [^18^F]Fluorobenzaldehyde ([^18^F]FBA) and *N*-[2-(4-[^18^F]-Fluorobenzamido)ethyl]maleimide ([^18^F]FBEM), makes radiofluorination of proteins possible in aqueous medium under mild conditions. [^18^F]SFB is a popular PG thanks to its reactivity with lysine residues, amino acid group naturally present on the surface of proteins, including sdAbs. Several [^18^F]FB-bioconjugates have demonstrated good in vivo stability, as shown by Kim et al. (2019); Jacobson et al. 2015; Gialleonardo et al. 2012; Bala et al. 2019; Xavier et al. 2016; Bala et al. 2016; Blykers et al. 2015). Distribution and commercialization of highly specific PET radiofluorinated radiopharmaceuticals becomes possible, while the centralized production of ^68^ Ga-labeled products is more difficult to organize (Wei et al. 2020; Vaidyanathan and Zalutsky 2006).

This study aims to develop a generic semi-automated radiofluorination strategy for sdAbs as a platform for the radiofluorination of two sdAb with high interest targets, namely FR-α and FAP-α. The production of the PG, [^18^F]SFB was optimized and automated on the AllInOne (AiO) module (Trasis), while the conjugation reaction to the sdAbs was achieved manually using an optimized protocol.

## Methods

The cell lines used in this study were generated for this purpose. The methodologies for their generation, culture conditions and validation by flow cytometry (supplemental Fig. 1) can be found in the Supplementary Information (SI).

### sdAbs

An anti-FAP-α sdAb, cross-reactive for mouse/human FAP-α and an anti-FR-α sdAb, reactive to human FR-α were kindly provided by Precirix. The anti-FAP-α sdAb (4AH29) (Dekempeneer et al. 2023), the FR-α sdAb (2BD42) and the non-targeting control sdAb (R3B23) (Lemaire et al. 2014) were produced and characterized as previously described (Broisat et al. 2012). All sdAbs in this study were free of tags.

### Radiochemistry

#### Automated [^18^F]SFB synthesis

**Fig. 1:**
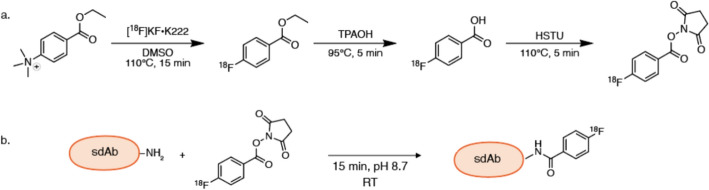
Synthesis of [^18^F]FB-sdAb: a. Synthesis of [^18^F]SFB in a three-step, one-pot reaction; b. conjugation of [^18^F]SFB to sdAb. RT = room temperature

*N*-succinimidyl-4-[^18^F] fluorobenzoate ([^18^F]SFB) was synthesized using a three-step, one-pot reaction (Fig. 1a). The complete production process of [^18^F]SFB, including the purification, was automated with an AiO module (Trasis) using disposable cassettes. [^18^F]F^−^ was produced by irradiation of enriched [^18^O]water (Rotem medical and Campro) in Niobium targets with a Cyclone KIUBE cyclotron (IBA) via the ^18^O(p,n)^18^F nuclear reaction. The [^18^F]fluoride aqueous solution was passed through a Sep-Pak Light Accell Plus QMA anion exchange cartridge (Waters) to trap [^18^F]fluoride and recover the enriched water. The [^18^F]fluoride was eluted from the cartridge with 600 µL of Cryptant Solution (4.2 mg of K_2_CO_3_ and 22.6 mg of Cryptand (K_222_) in acetonitrile/water (1:1)) (ABX). The solvent was evaporated to form anhydrous Kryptofix K_222_/K[^18^F]F complex (60–70 GBq). A solution of 0.8 mg (0.002 mmol) of ethyl-4-(trimethylammonium)benzoate (ABX) in 2 mL of dimethyl sulfoxide (DMSO) (Sigma-Aldrich) was added to the dried [^18^F]F^−^ complex in the reactor and heated to 110 °C for 15 min to produce ethyl-4-[^18^F]fluorobenzoate. This compound was hydrolysed at 95 °C for 5 min by a 0.38 M (0.76 mmol) tetrapropylammonium hydroxide (TPAOH) aqueous solution diluted in DMSO. The subsequent activation was performed with 26 mg (0.072 mmol) of *N*,*N*,*N*′,*N*′-tetramethyl-O-(*N*-succinimidyl)uronium hexafluorophosphate (HSTU, Sigma-Aldrich) in 1 mL of acetonitrile at 110 °C for 5 min to form [^18^F]SFB. The reaction mixture (RM) was diluted with 12 mL of an acetic acid solution (1.7% acetic acid/ NaCl 0.6%) before trapping on an HLB prime Plus Light solid-phase extraction (SPE) cartridge (Waters). The cartridge was washed with 1 mL of aqueous EtOH solution (5%) and reverse eluted with 0.8 mL of EtOH (Emsure, VWR). The purity of the [^18^F]SFB was determined by Reverse Phase High Performance Liquid Chromatography (RP-HPLC). Detailed information on the chromatographic analysis can be found in the SI.

The PG production described above and its automation was optimized based on the work of Xavier et al. (2016). Detailed insights into the optimization procedures are available in the SI, supplemental Table 1.

### Manual conjugation of [^18^F]SFB to sdAbs

At this point in the production of the tracers, the conjugation step was optimised and performed manually. The different sdAbs in phosphate buffered saline (PBS) pH 7.4 ± 0.1 (Table 1) are diluted with 0.5 M 2-(Cyclohexylamino)ethane-1-sulfonic acid (CHES) buffer pH 8.7 ± 0.1 and PBS. This mixture is added to 200 µL of the ethanolic (100%) [^18^F]SFB (3–5 GBq) and left to incubate for at least 15 min at room temperature (Fig. 1b). The radiolabelled sdAb was purified using two disposable desalting Hitraps (Cytiva) placed in series (pre-equilibrated with NaCl 0.9% with 5 mg/mL ascorbic acid, pH 5.9–6.2) using a peristaltic pump (Ismatec Reglo ICC, Masterflex) with a flow rate of 5 mL.min^−1^. The final product was passed through a 0.22 µm filter (Millipore) and analysed by RP-HPLC and Size-Exclusion (SE) -HPLC (see SI). Detailed insights into the optimization procedures, starting from the work of Xavier et al. (2016) are available in the SI, supplemental Table 2.

**Table 1:** Molecular weight and mass of sdAb used in conjugation reaction

sdAb	Target	Molecular weight (g/mol)	Amount of sdAb
4AH29	Mouse/human FAP-α	12,350.8	8.1 × 10^–8^ mol, 1000 µg, 100 µL
2BD42	FR-α	13,042.4	7.7 × 10^–8^ mol, 1000 µg, 100 µL
R3B23	Non targeting control	13,913.3	7.2 × 10^–8^ mol, 1000 µg, 100 µL

### Animal models

To facilitate the evaluation of off-tumour human FRα expression and biodistribution and tumour uptake of [^18^F]FB-2BD42, human FRα knock-in C57BL/6 transgenic mice were developed, as the designed radiotracer does not react with mouse FRα. These mice were produced by Cyagen (California, USA), and breeding took place at InnoSer (Leiden, The Netherlands). In summary, hFRα cDNA was inserted into exon 4 of the mFRα gene on chromosome 7 through homologous recombination, interrupting mFRα expression and enabling hFRα expression under the control of the native mFRα promoter. The targeting vector was electroporated into C57BL/6N embryonic stem cells, with neomycin selection used to isolate clones. The confirmed genotype was then injected into C57BL/6 albino blastocysts and implanted into pseudo-pregnant CD-1 females. Wildtype C57BL/6 female mice (Charles River) were used to evaluate biodistribution and tumour uptake of [[^18^F]FB-4AH29.

The ethical committee for animal experiments at the Vrije Universiteit Brussel approved the in vivo study protocols (22-272-12 & 19-272-17). They were subcutaneously inoculated at the tail base, under the control of 2.5% isoflurane in oxygen (Abbott), with TC-1-hFR-α cells (5 × 10^4^) suspended in PBS in the case of hFR-α knock-in mice and with TC-1-hFAP-α cells (5 × 10^4^) suspended in PBS in the case of the wildtype C57BL/6 mice. The tumours were allowed to grow for up to 2 weeks (100–300 mm^3^).

### Biodistribution & PET/CT imaging

hFR-α knock-in female mice bearing TC-1-hFR-α tumours (n = 4 per group) were i.v. injected (25 µg; 15 MBq) with [^18^F]FB-2BD42 or [^18^F]FB-R3B23. Wildtype C57BL/6 female mice bearing TC-1-hFAP-α tumours were i.v. injected (25 µg; 15 MBq) with [^18^F]FB-4AH29 (n = 4) or [^18^F]FB-R3B23 (n = 3). One hour after injection, micro-PET/CT images were acquired (detailed information in SI), followed by dissections 1h10 or 1h30 post injection in mice bearing TC-1-hFR-α tumours and mice bearing TC-1-hFAP-α tumours, respectively. The timepoint discrepancies are due to differences in the preclinical study design of both tracers. Animals were dissected, and organ and tissue activities were counted against a standard of known activity with an automated gamma counter (Wizard 2 2480, PerkinElmer) and expressed as a percentage of injected activity per gram (%IA/g), corrected for decay. In vitro characterization of the tracers, affinity measurement by cell saturation assay (supplemental Fig. 2) and in vitro stability in plasma (supplemental Table 3), can be found in the SI.

### Statistical analysis

Data were expressed as average ± SD. The statistical analysis used GraphPad Prism 10. One-way ANOVA, two-way ANOVA with multiple comparison tests, or unpaired t-test were used to evaluate statistical significance.

## Results

### Radiolabelling

[^18^F]SFB was synthesized using a three-step, one-pot reaction, which was fully automated. The total time of the procedure was 54 min and allowed to obtain [^18^F]SFB (23.31 ± 6.28 GBq, n = 13) with a RCP > 90% and a radiochemical yield (RCY) decay corrected (d.c.), corrected to the trapping of [^18^F]F^−^ on the QMA, of 44 ± 4% (n = 13).

**Fig. 2:**
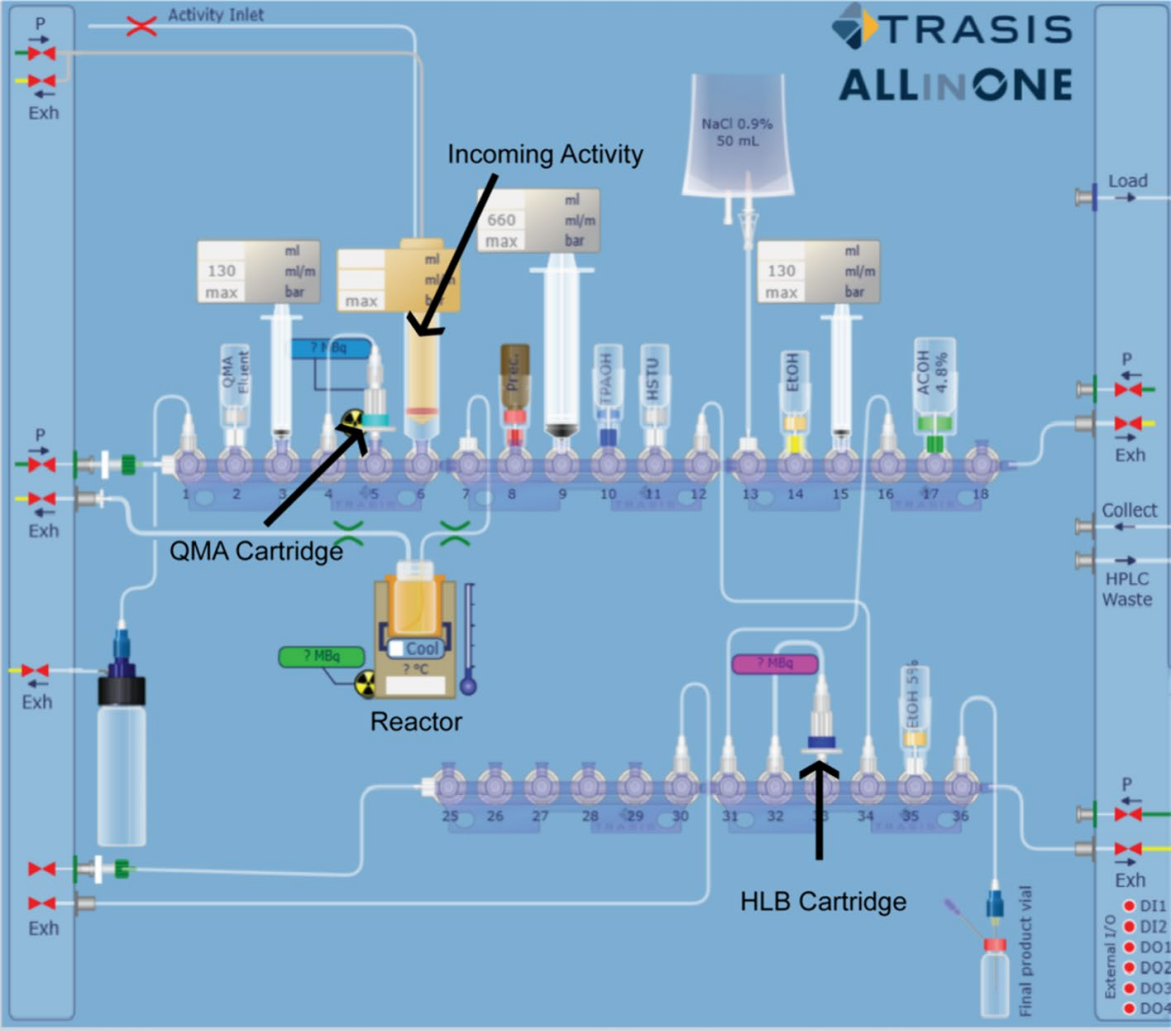
Layout of the automated radiosynthesis of [^18^F]SFB on a Trasis AiO. The 3-step one-pot procedure (upper row, rotors 117), as well as the purification of the PG (lower row and vial P14 and syringe P15), is included on the module

A schematic representation of the automated radiosynthesis procedure is shown in Fig. 2. The [^18^F]F^−^ enters the module via the syringe in the 6th position (P6) in the layout. The cyclotron-produced [^18^F]F^−^ is separated from the ^18^O-enriched water by the QMA cartridge on P5. Then, [^18^F]F is eluted with the Cryptand solution (P2), with the help of a syringe located in P3. The mixture is transferred to the 6 mL reactor (P7), after which the azeotropic drying of the [^18^F]fluoride is started. To the dried [^18^F]K_222_-fluoride, 0.8 mg of FB-precursor, dissolved in DMSO (P8), is added. The reactor is heated to 110 °C for 15 min to obtain the ethyl-4-[^18^F]fluorobenzoate and cooled down afterwards. Next, the product is hydrolysed by adding the 0.38 M TPAOH DMSO solution (vial P10) to the reactor. The reactor is heated to 95 °C for 5 min to obtain the 4-[^18^F]fluorobenzoic acid and cooled down again. For the third and last step, 26 mg of HSTU dissolved in anhydrous acetonitrile (P11) is transferred to the reactor. The reactor is heated to 110 °C for 5 min, obtaining the crude [^18^F]SFB, and cooled down again. The RM inside the reactor is diluted with a mixture of 4 mL of 4.8% acetic acid solution (P17) and 8 mL of 0.9% NaCl (P13), prepared by the module by mixing both components within the 20 mL syringe (P9) in the layout. The same syringe applies the RM to the HLB light cartridge (P33). Next, the cartridge and lines are rinsed with 5% EtOH/water solution (P35). To complete the purification, the final product is reverse eluted with EtOH (vial P14), using the 3 mL syringe (P15) and collected in a final vial.

The manual conjugation reaction produced [^18^F]FB-sdAbs with a RCY. of 22 ± 4% (n = 2), 19 ± 7% (n = 3) and 19 ± 1% (n = 2) d.c. (reference time for d.c. was the addition of [^18^F]SFB to conjugation mixture) for [^18^F]FB-2BD42, [^18^F]FB-4AH29 and [^18^F]FB-R3B23 respectively. The purified [^18^F]FB-sdAbs were obtained with a RCP > 95%, and the end of synthesis activity amounted to 783 ± 8.50 MBq (n = 2) for [^18^F]FB-2BD42, 694 ± 80 MBq (n = 2) [^18^F]FB-4AH29, and 907 ± 227 MBq (n = 2) for [^18^F]FB-R3B23. The apparent molar activity was 12.55 ± 0.21 GBq/µmol (n = 2), 10.42 ± 1.28 GBq/µmol (n = 2), and 15.58 ± 3.90 GBq/µmol (n = 2) respectively. Overall RCY d.c., corrected to the trapping of [^18^F]F^−^ on the QMA, were 9% (n = 1), 5 ± 2% (n = 3) and 8 ± 1% (n = 2) for [^18^F]FB-2BD42, [^18^F]FB-4AH29 and [^18^F]FB-R3B23 respectively.

### Biodistribution studies and PET/CT imaging

hFR-α knock-in female mice bearing TC-1-hFR-α tumours (n = 4 per group) were i.v. injected with [^18^F]FB-2BD42 (28 ± 2 µg; 14.69 ± 0.36 MBq, 6.95 ± 1.19 GBq/μmol) or [^18^F]FB-R3B23 (non-targeting control sdAb conjugate) (28 ± 2 µg; 16.08 ± 0.30 MBq, 8.13 ± 1.68 GBq/μmol). Wildtype C57BL/6 female mice bearing TC-1-hFAP-α tumours were i.v. injected with [^18^F]FB-4AH29 (26 ± 3 µg; 14.53 ± 1.33 MBq, 7.53 ± 0.88 GBq/μmol, n = 4) or [^18^F]FB-R3B23 (non-targeting control sdAb conjugate) (20 ± 0 µg; 12.75 ± 1.68 MBq, 8.68 ± 1 0.20 GBq/μmol, n = 3). Injected and apparent molar-specific activities are reported at the time of injection.

**Fig. 3:**
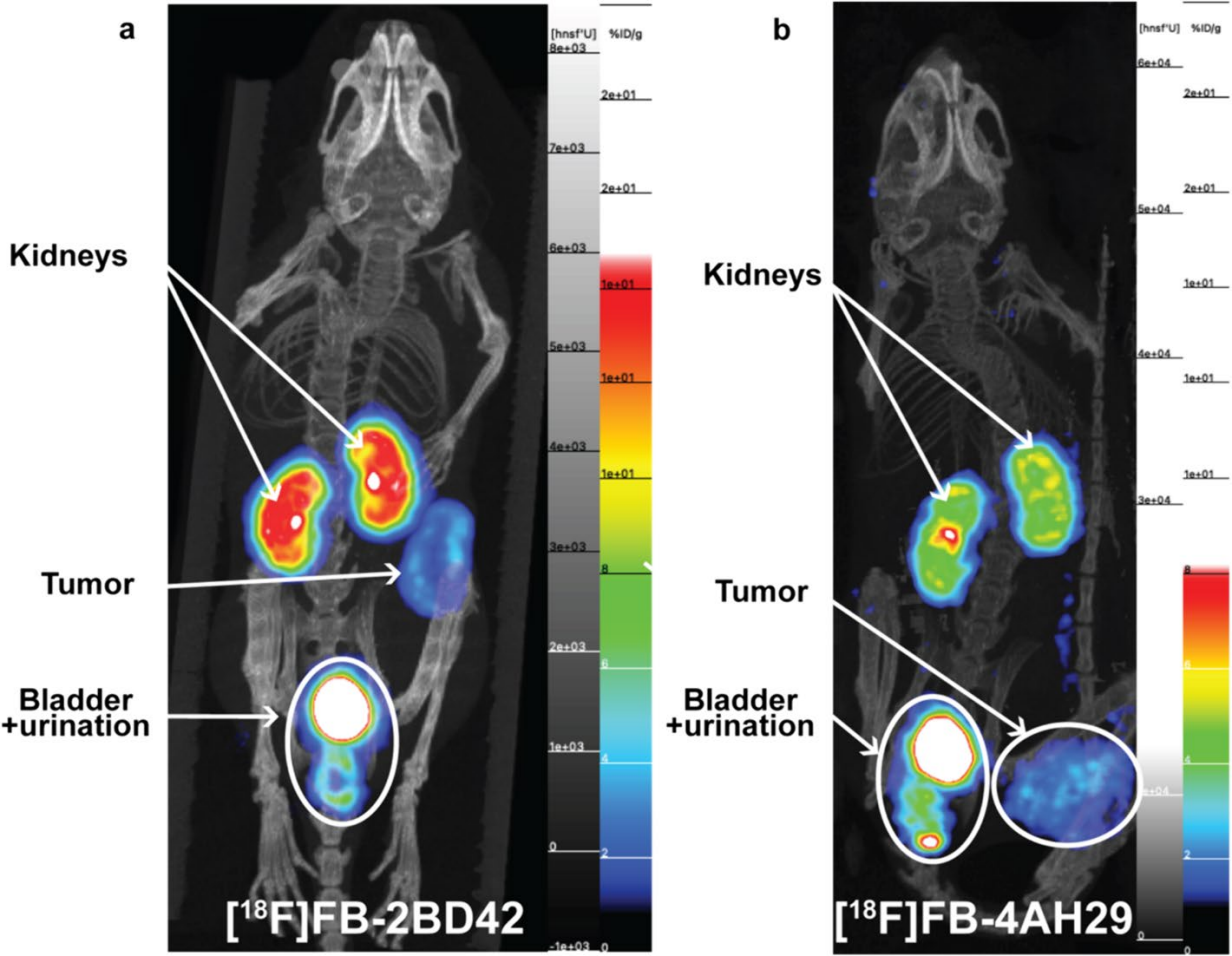
Maximum intensity projection PET/CT imaging of **a** [^18^F]FB-*2BD42* hFR-α knock-in mouse bearing TC-1-hFR-α tumours and **b** [^18^F]FB-4AH29 C57BL/6 mouse bearing TC-1-hFAP-α tumours 1 h p.i

**Fig. 4:**
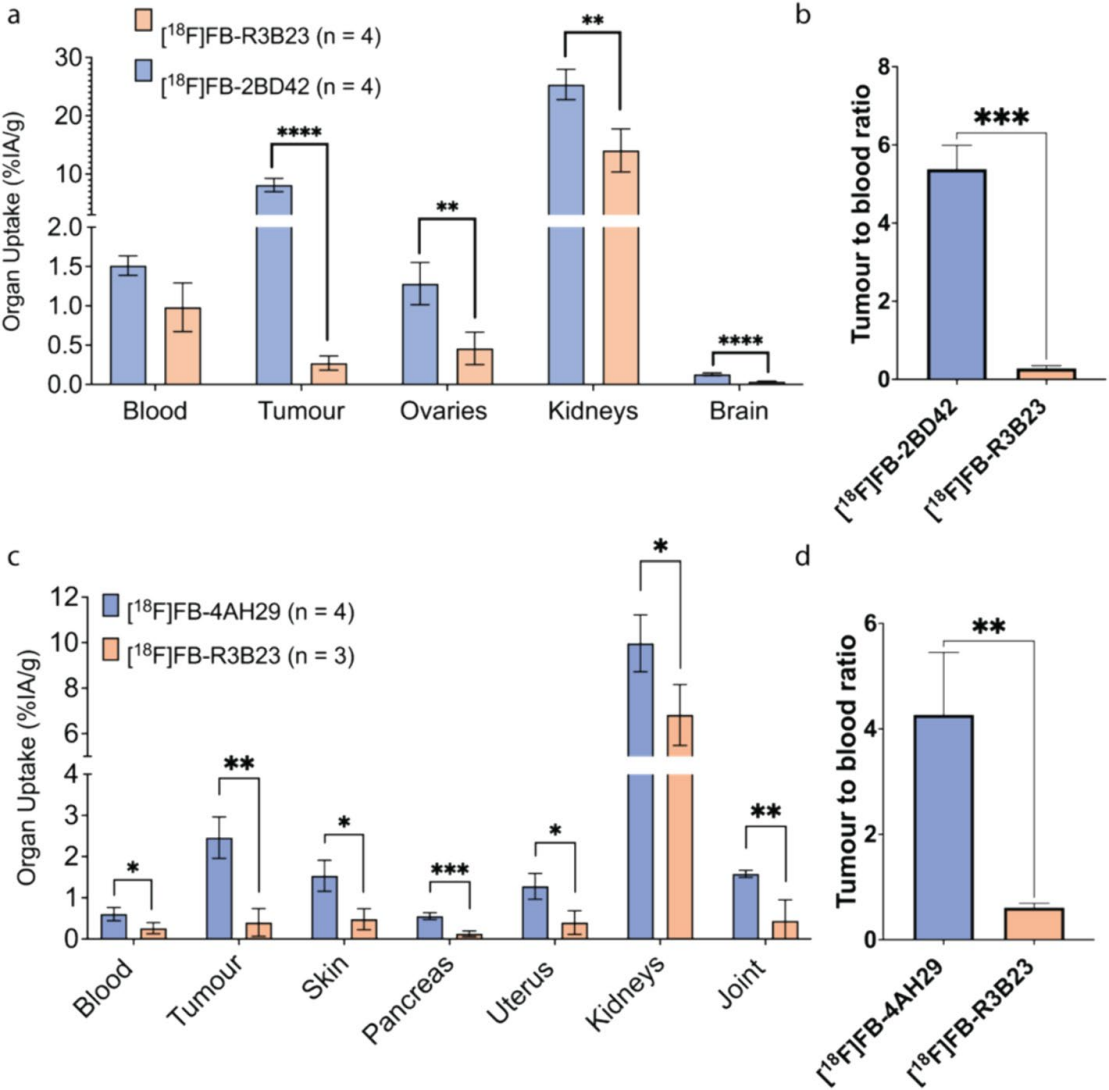
Ex vivo biodistribution results and T/B ratios of (i) [^18^F]FB-2BD42 compared to [^18^F]FB-R3B23 (A and B), 1h10 post injection; (ii) [^18^F]FB-4AH29 compared to [^18^F]FB-R3B23 (C and D) at 1h30 post injection. Two-way ANOVA or unpaired student t-test was used to calculate statistical significance. Statistical significance was set at *p* < 0.05 (ns, not significant, * *p* < 0.05; ** *p* < 0.01; *** *p* < 0.001; **** *p* < 0.0001)

Tumour uptake of [^18^F]FB-2BD42 was visible on the PET image (1 h p.i., Fig. 3a). It was confirmed by quantification of dissection data (1h10 p.i.) (Fig. 4a), showing statistically significant (*p* < 0.0001) higher tumour uptake (8.13 ± 1.15 IA/g) for the FR-targeting sdAb compared to the non-targeting sdAb (0.27 ± 0.09 IA/g). Furthermore, the dissection studies evaluating [^18^F]FB-2BD42 displayed about twofold higher kidney accumulation (25.37 ± 2.61 vs 14.06 ± 3.70 IA/g; *p* < 0.01), threefold higher accumulation in the ovaries (1.28 ± 0.27 vs 0.46 ± 0.21 IA/g; *p* < 0.01) and threefold higher accumulation in the brain (0.13 ± 0.02 vs 0.04 ± 0.01 IA/g; *p* < 0.0001) compared to the non-targeting sdAb. More detailed data concerning all measured organs can be found in SI, Supplemental Fig. 3a and Supplemental Table 4.

The in vivo profile of the anti-FAP-α sdAb, [^18^F]FB-4AH29, was investigated in TC-1-hFAP-α tumour bearing mice by a similar protocol, including micro-PET/CT imaging at 1 h p.i. (Fig. 3b) and dissection analysis at 1.5 h p.i. (Fig. 4c), and compared to [^18^F]FB-R3B23. Ex vivo biodistribution studies indicated specific tumour uptake (2.46 ± 0.50 IA/g) compared to the non-targeting sdAb (0.40 ± 0.34 IA/g), no unspecific organ accumulation except in the joints (1.58 ± 0.09 vs 0.44 ± 0.51 IA/g; *p* < 0.005), pancreas (0.55 ± 0.08 vs 0.13 ± 0.07 IA/g; *p* < 0.001), skin (1.53 ± 0.38 vs 0.48 ± 0.26 IA/g; *p* < 0.05), blood (0.60 ± 0.16 vs 0.26 ± 0.14 IA/g; *p* < 0.05) and uterus (1.28 ± 0.31 vs 0.40 ± 0.27 IA/g; *p* < 0.05) compared to the non-targeting sdAb. For both tracers, fast excretion of the unbound tracer was observed via the kidneys ([^18^F]FB-4AH29: 9.97 ± 1.25% IA/g; [^18^F]FB-R3B23: 6.82 ± 1.34% IA/g). More detailed data concerning all measured organs can be found in SI, Supplemental Fig. 3b and Supplemental Table 5.

The tumour-to-blood (T/B) ratios were calculated for both tracers. T/B ratios for [^18^F]FB-2BD42 and [^18^F]FB-4AH29 were significantly higher compared to their respective control sdAb (Fig. 4b, d).

## Discussion

The radiofluorination strategy of sdAbs described herein uses the well-established PG [^18^F]SFB. This PG is widely used for labelling peptides and proteins and its radio-synthesis has been continuously refined and optimized. In this study, the three-step, one-pot reaction was automated on a Trasis AiO. Automation of the PG production has been successfully implemented on in-house developed automation synthesis equipment (Fujimoto et al. 2021) and commercial automated synthesis modules such as the IBA Syntera module (Xavier et al. 2016; Blykers et al. 2015; Ackermann et al. 2011), TRACERlab FX_FN_ synthesizer (Scott and Shao 2010; Tang et al. 2010) (GE Healthcare) and the Ora-Neptis synthesizer (Nagachinta et al. 2022). We first optimized the automated production process by five times reducing the mass of the commercially available precursor (Xavier et al. 2016; Bala et al. 2016; Ackermann et al. 2011) without negatively impacting the RCY of the reaction (see SI, Table 1). We hypothesized that this reduction would also reduce the formation of potential process-related impurities and help increase specific activity. A second optimization was the purification of the PG. In the literature, different strategies can be found, such as HPLC methods, SPE using one single cartridge (Xavier et al. 2016; Ackermann et al. 2011; Scott and Shao 2010), multiple cartridges in series (Tang et al. 2010) or strategies combining both HPLC and SPE (Fujimoto et al. 2021; Nagachinta et al. 2022). The automated synthesis procedure described in this study uses a single SPE cartridge for purification, reducing time spent on purification compared to HPLC purification strategies. By opting for reverse elution of the cartridge, it was possible to reduce the elution volume to 800 µL. When comparing the SPE strategy used here to the other SPE strategies in literature (Xavier et al. 2016, 2019; Ackermann et al. 2011; Scott and Shao 2010; Tang et al. 2010), the final formulation of the PG in a small volume (0.8 mL) of ethanol, avoiding a reformulation step or time-consuming evaporation step before starting the subsequent conjugation reaction, is a significant advantage to reduce the time of the whole production process. The conjugation reaction described in this study was optimized with sdAbs in mind and included a 20% V/V content of ethanol. This ethanol concentration showed no negative impact on the conjugation reaction (see SI Table 2) and is in line with the results of several studies (Nikolaidis and Moschakis 2018; Nikolaidis et al. 2017) that showed denaturation of proteins caused by alcohols occurs at concentrations above 20%. The change of final solvent to ethanol was facilitated by replacing the previously used tC18 (Xavier et al. 2016; Bala et al. 2016; Ackermann et al. 2011) with an HLB cartridge. A slight reduction in RCP, > 90% compared to the previously reported (Xavier et al. 2016; Vaidyanathan and Zalutsky 2006; Scott and Shao 2010; Tang et al. 2010, 2008; Thonon et al. 2011) > 95%, could be observed, with [^18^F]FBA as the identified radioactive impurity. Most likely, this reduction in RCP is caused by a combination of hydrolysis, as the impurity increases over time, and radiolysis, increasing amount of radioactive impurity with increased volumetric activity concentration (up to more than 25 GBq/mL) and the observation became more apparent with upscaling of the reaction (see SI, supplemental Table 1). However, as the impurity does not compete with the PG in the following conjugation reaction, the slight decrease in RCP was deemed insignificant.

For optimization of the conjugation reaction, we opted for CHES as a coupling buffer due to the superior stability of the PG in this buffer compared to the conventional borate buffer (Xavier et al. 2016; Bala et al. 2016; Blykers et al. 2015). Nagachinta et al. (2022) performed the coupling of sdAbs to the PG using a phosphate buffer at pH 8.4, we prefer the use of CHES as its buffering range (pH 8.6–10 compared to 5.8–7.4 for a phosphate buffer) is more in range with the optimal reactivity of the sdAbs’ amino groups towards acylation (pH < 8.5). The higher buffer capacity and, thus fewer fluctuations in pH of CHES compared to phosphate also allows for a more robust coupling reaction. Detailed insights into the buffer selection are available in the SI, supplemental Fig. 4. The purification of the radiolabelled sdAbs was performed using SE resins HiTrap desalting cartridges instead of the PD-10 desalting column, with the latter being the most described option in literature (Xavier et al. 2016, 2019; Bala et al. 2016; Blykers et al. 2015; Nagachinta et al. 2022). The main advantage of these cartridges compared to gravity-based SEC is their compatibility with the manifolds of our automation module, making it a plug-and-play approach. While gravity-based cartridges, like PD-10 columns could be implemented in an automated production (Nagachinta et al. 2022), they do require an auxiliary device. The conjugation of the sdAbs to the PG resulted in reasonable decay-corrected conjugation yields (20–25%, starting from [^18^F]SFB) with high RCP and reasonable apparent molar activity. The conjugation yield was comparable to or higher than others reported for sdAbs and proteins (Xavier et al. 2016; Bala et al. 2016; Scott and Shao 2010; Nagachinta et al. 2022; Thonon et al. 2011; Davis et al. 2019). The comparable conjugation results (similar RCY d.c., apparent molar activities. and final activities) for all three sdAbs show that this strategy could also be used as a generic radiolabelling strategy for sdAbs, similar to the generic ^68^Ga-chelator approach currently used (Keyaerts et al. 2016; Gondry et al. 2024, 2023; Dekempeneer et al. 2023; Xavier et al. 2019). This generic ^68^ Ga-chelator approach has already been successfully used to introduce sdAb-based tracers in the clinic, as shown by the clinical translation of sdAbs targeting HER2 (Keyaerts et al. 2016; Gondry et al. 2024) and CD206 (Xavier et al. 2019; Gondry et al. 2023). The advantages of this method compared to radiofluorination are the ease of its chemistry, higher RCYs and its lower initial financial investment, as there is no need for a cyclotron or automation modules. On the other hand, by developing a radiolabelling method with ^18^F for sdAbs, we can take advantage of the superior imaging quality of ^18^F. At the same time, its longer half-life allows for easier radiopharmaceutical distribution and still matches the biological half-life of sdAbs. Because of the ease of production of high amounts of the radionuclide with a cyclotron, upscaling the obtained activity will allow for multi-patient preparations produced in PET radiopharmacies or centralized production sites.

The biodistribution and imaging studies for both tracers showed excellent targeting properties and specificity for FR-α or FAP-α, fast excretion via the kidneys of both [^18^F]FB-2BD42 and [^18^F]FB-4AH29, respectively. The known FR-α expression in the fallopian tubes, proximal tubule cells of the kidneys, and choroid plexus in the brain, might explain the observed elevated uptake in these organs (Scaranti et al. 2020; Boss and Ametamey 2020; Parker et al. 2005).

Besides specific uptake of [^18^F]FB-4AH29 in the tumour, elevated accumulation was seen in pancreas, skin and uterus. This is in line with previous findings (Li et al. 2012; Keane et al. 2014) in mice, showing an interspecies difference in FAP expression compared to humans. The elevated uptake in blood and joints could be attributed to the increased shedding of FAP protein in mice (Keane et al. 2014), while the elevated uptake in the joints to FAP expression of murine multipotent bone marrow stromal cells (Chung et al. 2014).

## Conclusion

Using a Trasis AiO, [^18^F]SFB synthesis was successfully automated and upscaled, yielding consistently around 20 GBq of pure product. The anti-hFAP-α, anti-hFR-α and non-targeting control sdAbs were successfully radiofluorinated, yielding similar RCYs d.c. and RCPs. The herein presented semi-automated radiofluorination approach could be used as a generic radiofluorination method for sdAbs, allowing for faster preclinical validation of sdAbs as PET tracers and opens opportunities for further development towards clinical production. The radiofluorinated sdAbs showed a favourable biodistribution pattern and are attractive for further characterization as new PET tracers for FAP-α and FR-α imaging.

### Supplementary Information

Additional file 1. Supplementary figures and tables.

## Data Availability

The datasets generated and/or analysed during the current study are available from the corresponding author on reasonable request.

## Abbreviations

[^18^F]FBA: [^18^F]Fluorobenzaldehyde
[^18^F]FBEM: *N*-[2-(4-[^18^F]-Fluorobenzamido)ethyl]maleimide
[^18^F]SFB: *N*-Succinimidyl 4-[^18^F]Fluorobenzoate
^18^F: Fluorine-18
^68^ Ga: Gallium-68
AiO: AllInOne
CHES: 2-(Cyclohexylamino)ethane-1-sulfonic acid
d.c.: Decay corrected
DMSO: Dimethyl sulfoxide
EtOH: Ethanol
FAP: Fibroblast activation protein
FR: Folate receptor
HSTU: *N*,*N*,*N*′,*N*′-Tetramethyl-O-(*N*-succinimidyl)uronium hexafluorophosphate
mAbs: Monoclonal antibodies
PBS: Phosphate buffered saline
PG: Prostethic group
RCP: Radiochemical purity
RCY: Radiochemical yield
RM: Reaction mixture
RP-HPLC: Reverse phase high performance liquid chromatography
sdAbs: Single-domain antibodies
SE: Size-exclusion
SI: Supplementary information
SPE: Solid-phase extraction
T/B: Tumour-to-blood
TPAOH: Tetrapropylammonium hydroxide
