# Supplementary material for: Generic semi-automated radiofluorination strategy for single domain antibodies: [18F]FB-labelled single domain antibodies for PET imaging of fibroblast activation protein-α or folate receptor-α overexpression in cancer
Source: EJNMMI Radiopharm Chem. 2024 Jul 24;9:54. doi: 10.1186/s41181-024-00286-8 (PMC11269545; doi:10.1186/s41181-024-00286-8)
Supplement: Supplementary file 1 — Additional file 1. Supplementary figures and tables. [file 41181_2024_286_MOESM1_ESM.docx]

### Chromatographic analysis

Size-exclusion high performance liquid chromatography (SE-HPLC) analysis was performed using a Superdex 75 Increase 5/150 column (Cytiva) and 0.01 M phosphate buffer with 0.14 M NaCl (PBS), pH 7.4, at a flow rate of 0.45 mL.min−1. The SE-HPLC analyses were performed on a Merck Hitachi Chromaster system (VWR) connected to a diode array detector and γ-detector (Raytest).

Reverse phase high performance liquid chromatography (RP-HPLC) analysis was performed using a diisopropylcyanopropylsilane column 160 Å ES-CN 2.7 µM. 4.6 x 150 mm (HALO) and the following gradient (A: 0.1% trifluoroacetic acid in water; B: 0.1% trifluoroacetic acid in acetonitrile): 0–1.5 min. 30% B; 1.5–3 min. 30%-100% B; 3-4.5 min. 100% B; 4.5-5 min 100%-30% B; 5-8 min 30% B; at a flow rate of 1.6 mL.min−1.The column was heated to a temperature of 40°C. The RP-HPLC analyses were performed on a Shimadzu Prominence LC-40AT system connected to a UV-VIS detector and γ-detector (Elysia- Raytest).

### PET/CT imaging

The acquisition of the PET and CT images were respectively carried out with a β-CUBE PET system and a X-CUBE CT system (both from MOLECUBES), 1 h p.i. The total PET/CT scanning time was 6 minutes. The PET images were acquired over 5 min and reconstructed into a matrix of 193 × 192 × 384 voxels with 400 μm voxel size. The CT images were iteratively reconstructed using the ISRA reconstruction algorithms into 200 μm voxels (matrix 200 × 200 × 393).

### Cell line generation

#### Parental cell lines and culture conditions

Human embryonal kidney (HEK) 293T cells (CRL-3216) were purchased from the American Type Culture Collection and registered at the Vrije Universiteit Brussel biobank under the study code B084. HEK293T cells were cultured at 37°C, 5% CO_2_ and 95% humidity in Dulbecco’s Modified Eagle Medium containing 10% fetal bovine serum (TICO Europe), 100 units/mL penicillin, 100 µg/mL streptomycin and 2mM L-Glutamine. Mouse TC-1 lung epithelial cancer cells were provided by T.C. Wu (Johns Hopkins University, Baltimore, Maryland, USA) and cultured at 37°C, 5% CO_2_ and 95% humidity in Roswell Park Memorial Institute 1640 medium (Sigma-Aldrich) containing 10% fetal clone I serum (Thermo Fisher Scientific), 100 U/mL penicillin, 100 µg/mL streptomycin, 1 mM sodium pyruvate with nonessential amino acids, 2 mM L-Glutamine, 12.5 mM D(+)-glucose, 5 mM 4-(2-hydroxyethyl)-1-piperazineethanesulfonic acid, 50 µM β-mercaptoethanol and 1 mM Geneticin (G418). All culture media and supplements were purchased from Sigma-Aldrich unless noted otherwise.

#### Generation and characterization of lentiviral vectors

The pMD.G envelope plasmid and the pCMV∆R8.9 packaging plasmid were obtained from Didier Trono (University of Geneva, Switzerland). The pHR’ transfer plasmids with EF-1α promotor were modified to contain the genetic code for hFR-α or hFAP-α using the Gibson assembly method with gBlocks from Integrated DNA Technologies (IDT) that were designed to encode human FR-α (hFR-α) or human FAP-α (hFAP-α). All plasmid sequences were confirmed by sequence analysis (Eurofins). Lentiviral vectors were produced, and a 1000-times concentrated as previously described [1] Lentiviral titers were determined as previously described [2] with the following adaptations: HEK293T cells were harvested and stained with antibodies specific for hFR-α (No.5/FOLR, Biolegend) or hFAP-α (427819, R&D Systems) 3 days following transduction of HEK293T cells with a serial dilution of the lentiviral vectors. The frequency of transgene positive cells was determined for each dilution by flow cytometry with the acquisition of the cells on the LSR Fortessa flow cytometer (BD Biosciences) and analysis of the data via Flow Jo software version 10.0 (Tristar. Inc). The number of transducing units (TU) per millilitre (mL) lentiviral stock was determined using the following formula: {F × (C/V)} × D. Herein, F is the frequency of transgene positive cells, C is the total number of cells at the time of transduction, V is the volume of the transduction cocktail and D is the dilution used. The average of the TU/mL obtained per dilution was considered as the final titer.

#### Lentiviral transduction of TC-1 cells

Mouse TC-1 cells were transduced with hFR-α or hFAP-α encoding lentiviral vectors at 1×10^E5^ cells in 2 mL of culture medium at a multiplicity of infection of 5, generating hFR-α^POS^ or hFAP-α^POS^ TC-1 cells. To ensure >95% hFR-α or hFAP-α expression, hFR-α^POS^ or hFAP-α^POS^ TC-1 cells were enriched via fluorescence-activated cell sorting. To that end, cells were stained with antibodies specific for hFR-α (No.5/FOLR, Biolegend) or hFAP-α (427819, R&D Systems) and sorted on the BD FACSMelody (BD Biosciences). The hFR-α or hFAP-α expressions on the enriched and expanded hFR-α^POS^ or hFAP-α^POS^ TC-1 cells were evaluated following staining with the antibodies as mentioned earlier, the eFluor506 Fixable Viability dye (Invitrogen), acquisition of the samples on the LSR Fortessa (BD Biosciences), and data analysis using Flow Jo software version 10.0 (Tristar. Inc) (supplemental data; Figure 1).

#### Cells culture conditions of transduced cell lines

The murine tumour cell lines TC-1 hFR-α and TC-1 hFAP-α were cultured in RPMI1640 medium supplemented with 10% Fetal clone I serum (Thermo Scientific. Belgium), 2 mM *L*-Glutamine, 100 U/mL penicillin, 100 µg/mL streptomycin, 1 mM sodium pyruvate, and nonessential amino acids.

All cells were grown in monolayer in Falcon tissue culture dishes (Becton Dickinson. Franklin Lakes. NJ. USA) and incubated at 37°C in a humidified incubator with 5% CO_2_. Cells were detached with trypsin-EDTA in PBS (Invitrogen).

#### Flow cytometry validation of transduced cell lines


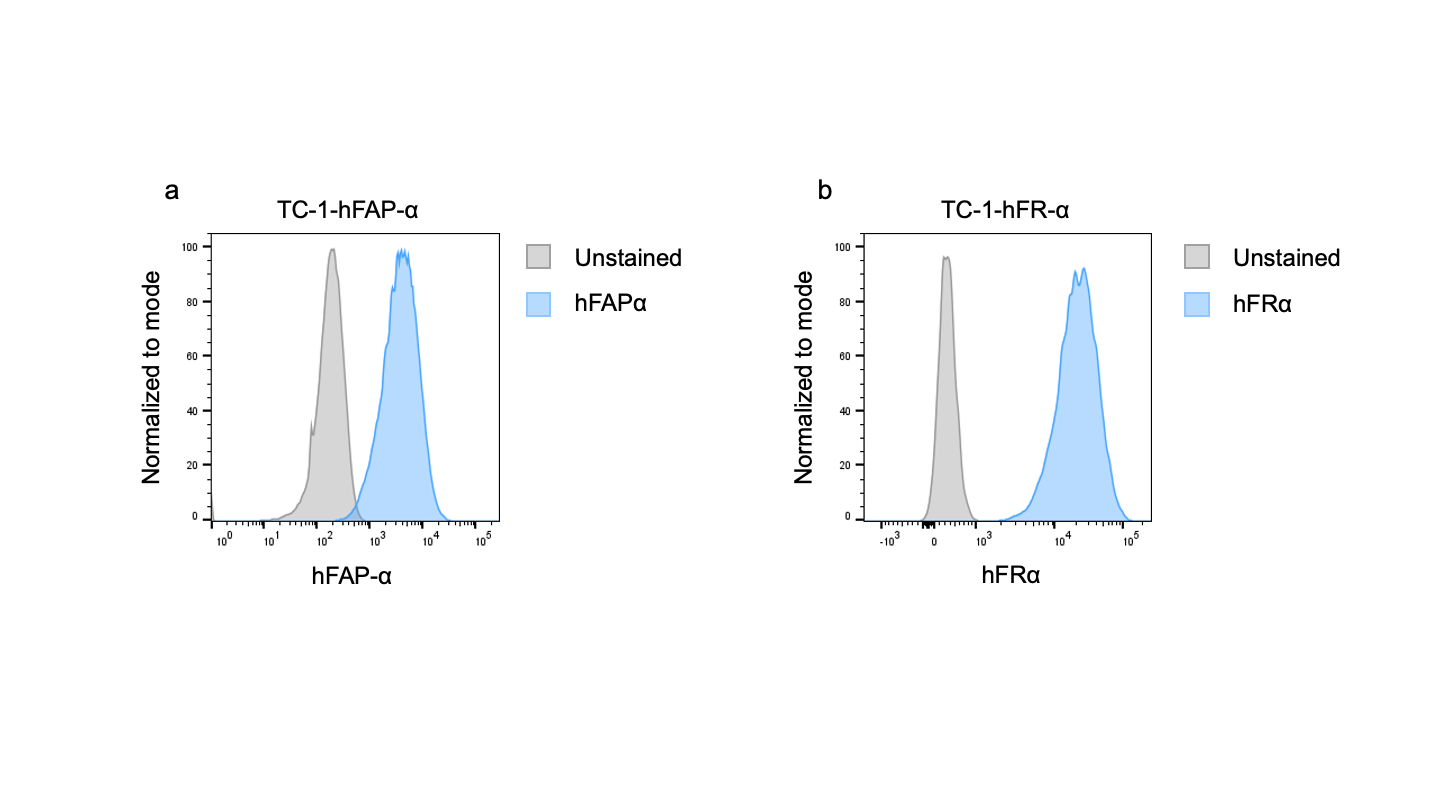


Supplemental Figure 1: Flow cytometry analysis of hFR-α (b) or hFAP-α (a) expressions on cell lines used in this study. Histogram plots showing FAP expression (blue) on the cell lines used in this study, via flow cytometry.

### Radiochemistry optimization

Table 1: Prosthetic group production: Automation on Trasis AiO, optimization & upscaling

|  | Conditions | RCP | RCY d.c. | Starting Activity |
| --- | --- | --- | --- | --- |
| 1 | Conditions Xavier et al.[3] on trasis AiO | > 95% | 16% | 2 Gbq |
| 2 | TPAOH dissolved in 0,5 mL DMSO | > 95% | 24% | 2,35 GBq |
| 3 | Change in preconditioning QMA: from 1.4% to 8.1% NaHCO3 | > 95% | 31% | 9 GBq |
| 4 | Upscaling | > 95% | 28% | ±15 Gbq |
| 5 | Reduction mass precursor | > 95% | 25% | 14 GBq |
| 6 | Upscaling | > 95% | 31% | 50 Gbq |
| 7 | Implementation HLB purification reverse elution | > 95% | 35% | 10 GBq |
| 8 | Upscaling | > 90% | 36% | ±65 Gbq |
| *9 | Increasing elution volume HLB | > 90% | 44% | ±65 Gbq |
| 10 | Upscaling | > 90% | 46% | ±100 Gbq |

*Process used and described in this article.

Table 2: Conjugation of [^18^F]SFB to sdAbs: optimization & upscaling

|  | Conditions | RCP | RCY d.c. | Starting Activity | Apparent Molar Activity (GBq/µmol) |
| --- | --- | --- | --- | --- | --- |
| *1 | Conditions Xavier et al. [3] (including sdAb) | > 95% | 18% | 338 MBq | Not calculated |
| *2 | - Increasing sdAb concentration: 1.5 mg/mL  - Phosphate buffer 0,2M pH 8,7  - Purification: NAP5 | > 70% | 21% | 431 MBq | Not calculated |
| 3 | - Increasing sdAb concentration: 5 mg/mL  - 20% Ethanol  - Phosphate buffer 0.2M pH 8.7  - Purification: NAP5 | > 95% | 24% | 352 MBq | 2,69 |
| 4 | Same conditions as 3 but 30 min reaction time & upscaling activity  Purification: PD-10 | > 95% | 22% | 4,73 GBq | 30,85 |
| 5 | Further upscaling | > 95% | 16% | 8,17 GBq | 33,79 |
| **6 | 20% EtOH  1.85 mg/mL sdAb  Reaction time: 15 min  Purification: 2 Hitraps in series | > 95% | 29% | ±1,5 GBq | > 0,5 |
| 7 | Reduction sdAb concentration: 1.5 mg/mL  Upscaling activity | > 95% | 19% | 5 GBq | 2,52 |
| 8 | Same conditions as before; different conjugation buffer CHES 0,1M  Reaction time 60 minutes | > 95% | 25% | 4,8 GBq | 6,70 |
| 9 | Reduction sdAb concentration: 1mg/mL  Reaction time: 15min- 30min | > 95% | 17% | 5 GBq | ≥ 10 |

*Experiments done with 2Rs15d [3], all other results mentioned in table 2 were obtained with 4AH29 and 2BD42.

**Entry 6 and later: New purification method PG implemented: PG already dissolved in pure ethanol

### In vitro characterization of [^18^F]FB-1012& [^18^F]FB-4AH29

#### Affinity Measure (K_D_) by Cell Saturation Assay

The affinity of the radiolabelled sdAbs towards FAP-α or FR-α was assessed on FAP-α- or FR-α-expressing transduced TC-1 cells. Two days before the experiment, 1.2x10^4^ cells in 1 mL of medium per well were allowed to attach to a 24-well plate at 37 °C. The plate was cooled to 4 °C 1 h before the experiment. The supernatant was removed, and the cells were incubated for 30 minutes at 4 °C with 500 μL of the [^18^F]FB-labelled sdAb solution at different concentrations ranging from 300 to 0.1 nM in an unsupplemented medium (n = 3 wells per condition). To correct for nonspecific binding, the same procedure was simultaneously applied to a second plate containing a 100-molar excess of each tested concentration of unlabelled sdAb in each well. The K_D_ was calculated using Prism software's “One site —total and nonspecific binding” analysis. Data were expressed as average ± SEM.

The dissociation constant (K_D_) was determined as 29.02 ± 3.77 nM for the [^18^F]FB-2BD42 and 15.86 ± 2.66 nM for [^18^F]FB-4AH29 (figure 2a & 2b).


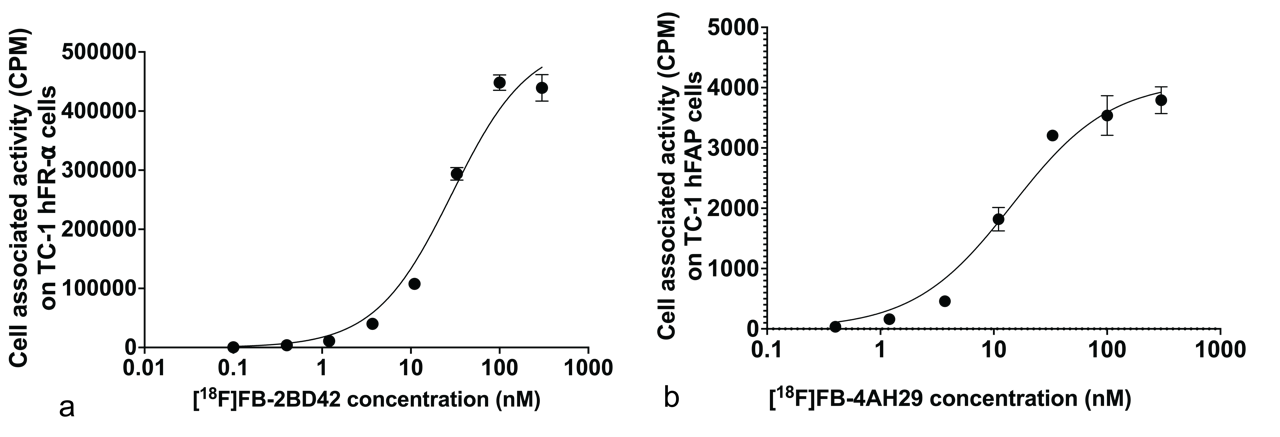


Supplemental figure 2: Radioligand binding study on TC-1 hFR-α cells (A) & TC-1 hFAP-α cells (B) showing specific binding curve. Cell bound activity in counts per minute (CPM) expressed as a function of the sdAb concentration (nM) for (A) [^18^F]FB-2BD42 and (B) [^18^F]FB-4AH29

#### In vitro stability of [18F]FB-1012 & [18F]FB-4AH29

The stability of [^18^F]FB-sdAbs (40-50 MBq) was evaluated in human serum at 37 °C during 2h. At different time points, samples were analysed using radio-SE-HPLC or radio-RP-HPLC for [^18^F]FB-2BD42 and [^18^F]FB-4AH29 respectively.

Both ^18^F-labeled sdAbs were stable over 2 h (RCP > 95%). No degradation of either probe was observed (Table 3).

Table 3: Stability expressed as radiochemical purity (RCP) of the [^18^F]FB-2BD42 and [^18^F]FB-4AH29 in human serum (HS° at 37 °C).

| Timepoint (minutes) | RCP [^18^F]FB-2BD42 | RCP [^18^F]FB-4AH29 |
| --- | --- | --- |
| 0 | > 99% | > 99% |
| 15 | 98,5% | Not measured |
| 60 | 96,6% | 96% |
| 120 | > 95% | 95% |

### Biodistribution studies and PET/CT imaging

Ex vivo biodistribution results including all organs (i) [^18^F]FB-2BD42 compared to [^18^F]FB-R3B23 (Supplemental Figure 3a and supplemental Table 4), 1h10 post injection; (ii) [^18^F]FB-4AH29 compared to [^18^F]FB-R3B23 (Figure 3b and supplemental Table 4) at 1h30 post injection


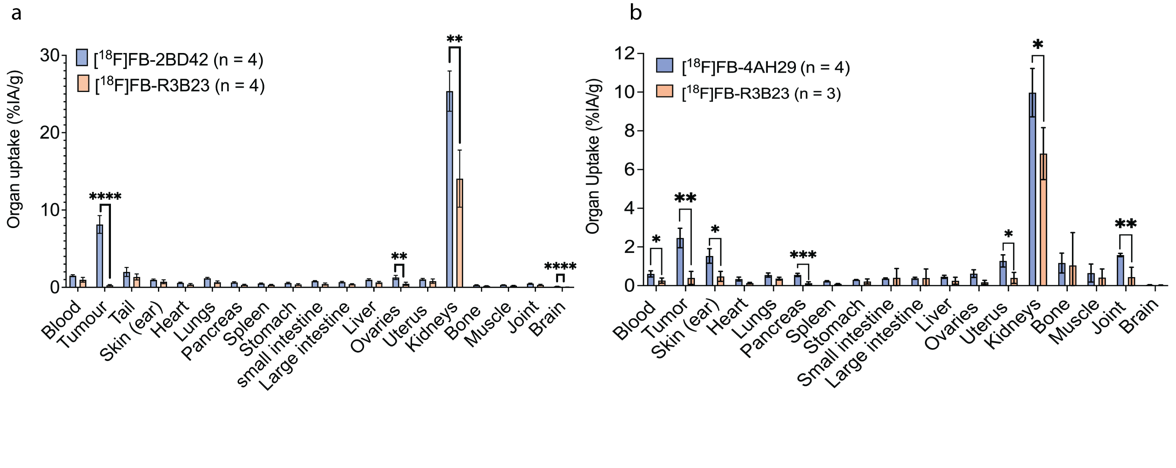


Supplemental Figure 3: Ex vivo biodistribution results including all organs (a) [^18^F]FB-1012 compared to [^18^F]FB-R3B23 1h10 post injection; (b) [^18^F]FB-4AH29 compared to [^18^F]FB-R3B23 at 1h30 post injection. Two-way ANOVA or unpaired student t-test was used to calculate statistical significance. Statistical significance was set at p<0.05 (ns, not significant, * p<0.05; ** p<0.01; *** p<0.001; **** p<0.0001).

Table 4: Ex vivo biodistribution results including all organs [^18^F]FB-1012 compared to [^18^F]FB-R3B23

|  | [^18^F]FB-2BD42 (n= 4) | | [^18^F]FB-R3B23 (n = 4) | |  |
| --- | --- | --- | --- | --- | --- |
| Blood | 1,5113 | 0,124 | 0,9819 | 0,3089 | |
| Tumor | 8,1321 | 1,1508 | 0,2725 | 0,0897 | |
| Tail | 1,9925 | 0,5789 | 1,3466 | 0,3868 | |
| Skin (ear) | 0,9639 | 0,1151 | 0,73 | 0,2517 | |
| Heart | 0,5938 | 0,0732 | 0,3749 | 0,1286 | |
| Lungs | 1,1753 | 0,1109 | 0,6671 | 0,1592 | |
| Pancreas | 0,6267 | 0,088 | 0,314 | 0,0809 | |
| Spleen | 0,4882 | 0,0743 | 0,3227 | 0,0631 | |
| Stomach | 0,5529 | 0,0954 | 0,3727 | 0,1077 | |
| small intestine | 0,8008 | 0,0701 | 0,4295 | 0,1351 | |
| Large intestine | 0,6736 | 0,0969 | 0,4043 | 0,0783 | |
| Liver | 0,9828 | 0,1279 | 0,6412 | 0,1345 | |
| Ovaries | 1,2819 | 0,2681 | 0,4596 | 0,2059 | |
| Uterus | 1,0358 | 0,13 | 0,8054 | 0,2854 | |
| Kidneys | 25,3713 | 2,60760258 | 14,0602 | 3,69620283 | |
| Bone | 0,284 | 0,0536 | 0,1768 | 0,0403 | |
| Muscle | 0,3172 | 0,0184 | 0,2169 | 0,0509 | |
| Joint | 0,48 | 0,0687 | 0,3207 | 0,0743 | |
| Brain | 0,1303 | 0,0176 | 0,0373 | 0,0077 | |

Table 5: Ex vivo biodistribution results including all organs [^18^F]FB-4AH29 compared to [^18^F]FB-R3B23

|  | [^18^F]FB-4AH29 (n= 4) | | [^18^F]FB-R3B23 (n = 3) | |
| --- | --- | --- | --- | --- |
| Blood | 0,6046 | 0,1602 | 0,2613 | 0,1354 |
| Tumor | 2,4605 | 0,5036 | 0,4009 | 0,3376 |
| Skin (ear) | 1,5339 | 0,3765 | 0,4795 | 0,2564 |
| Heart | 0,3385 | 0,1 | 0,1315 | 0,039 |
| Lungs | 0,5499 | 0,1062 | 0,3628 | 0,073 |
| Pancreas | 0,5545 | 0,0818 | 0,1268 | 0,069 |
| Spleen | 0,226 | 0,0301 | 0,0901 | 0,0203 |
| Stomach | 0,2974 | 0,0169 | 0,2176 | 0,1297 |
| Small intestine | 0,3626 | 0,0365 | 0,4086 | 0,4763 |
| Large intestine | 0,384 | 0,0529 | 0,3953 | 0,4676 |
| Liver | 0,4569 | 0,0817 | 0,2544 | 0,1798 |
| Ovaries | 0,6202 | 0,1972 | 0,1722 | 0,1041 |
| Uterus | 1,2779 | 0,3145 | 0,3996 | 0,2859 |
| Kidneys | 9,9693 | 1,24908947 | 6,8245 | 1,34213679 |
| Bone | 1,1682 | 0,511 | 1,0461 | 1,6906 |
| Muscle | 0,6513 | 0,467 | 0,418 | 0,4429 |
| Joint | 1,5808 | 0,0871 | 0,4414 | 0,5074 |
| Brain | 0,0455 | 0,006 | 0,0248 | 0,0141 |

### Stability of the PG in different potential conjugation buffers

The stability of the PG was tested in different possible couplings buffers, all with a pH between 8.5 and 8.7. The dried PG was dissolved in mixture of PBS, conjugation buffer to be tested and Ethanol. The concentration of the buffers was 0.1 M and 6% V/V ethanol in the mixture. The PG was degraded in a borate but remained stable in a potassium phosphate buffer as well as in CHES buffer.

Two polystyrene divinylbenzene copolymer columns, A (PLRP-S 300 Å, 5 μm, 250/4 mm) and B (PLRP-S 100 Å, 5 μm, 250/4 mm, Agilent) were used with the following gradient (A: 0.05% trifluoroacetic acid in water; B: 0.05% trifluoroacetic acid in acetonitrile): 0–7 min, 10%-100% B; 7–10 min, 100% B; 10-10.01 min, 100%-10% B, 10.01-15 min 10% B, at a flow rate of 1.25 mL min^−1^. Column A was used for the stability assessment of the PG in borate 0.1 M and potassium phosphate 0.1 M. Column B was used for the stability assessment of the PG in CHES 0.1 M. The analyses were performed on a Shimadzu Prominence LC-40AT system connected to UV-VIS detector and γ-detector (Elysia- Raytest, Germany).


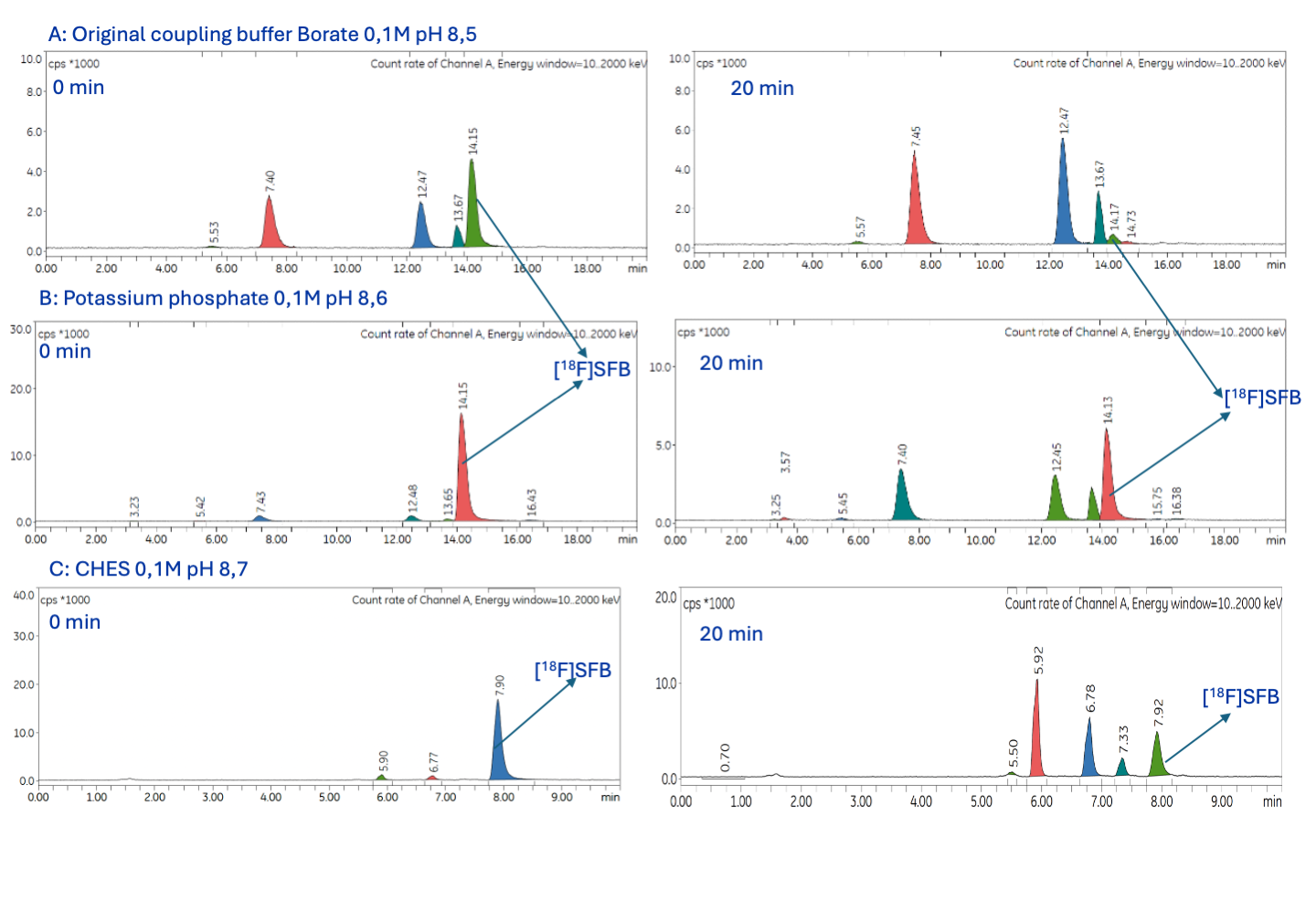


Supplemental Figure 4: Stability assessment of PG group in different conjugation buffers A: Original coupling buffer Borate 0.1 M pH 8.5: peaks with Rt= 5.53; 7.40; 13.67 min = unidentified peaks; peak Rt = 12.47 min = hydrolysed [^18^F]SFB; Peak Rt= 14.15 min = [^18^F]SFB ; B: Potassium phosphate 0.1 M pH 8.6: peaks with Rt= 3.25; 5.42; 7.40; 13.65; 15.75; 16.38 min = unidentified peaks; peak Rt = 12.45 min = hydrolysed [^18^F]SFB; Peak Rt= 14.15 min = [^18^F]SFB; C: CHES 0.1 M pH 8.7: peaks with Rt= 5.50; 5.92; 6.78; 7.33 min = unidentified peaks; Peak Rt= 7.90 min = [^18^F]SFB at timepoint 0 minutes and 20 minutes.

### References

1. Goyvaerts C, De Groeve K, Dingemans J, Van Lint S, Robays L, Heirman C, et al. Development of the Nanobody display technology to target lentiviral vectors to antigen-presenting cells. Gene Ther. 2012;19:1133–40.

2. Breckpot K, Dullaers M, Bonehill A, Van Meirvenne S, Heirman C, De Greef C, et al. Lentivirally transduced dendritic cells as a tool for cancer immunotherapy. J Gene Med 2003;5:654–67.

3. Xavier C, Blykers A, Vaneycken I, D’Huyvetter M, Heemskerk J, Lahoutte T, et al. 18F-nanobody for PET imaging of HER2 overexpressing tumors. Nucl Med Biol. 2016;43:247–52.
